# Biosynthesis of Sesquiterpene Lactones in Pyrethrum (*Tanacetum cinerariifolium*)

**DOI:** 10.1371/journal.pone.0065030

**Published:** 2013-05-31

**Authors:** Aldana M. Ramirez, Nils Saillard, Ting Yang, Maurice C. R. Franssen, Harro J. Bouwmeester, Maarten A. Jongsma

**Affiliations:** 1 Plant Research International, Wageningen University and Research Centre, Wageningen, The Netherlands; 2 Laboratory of Plant Physiology, Wageningen University and Research Centre, Wageningen, The Netherlands; 3 Laboratory of Entomology, Wageningen University and Research Centre, Wageningen, The Netherlands; 4 Laboratory for Organic Chemistry, Wageningen University and Research Centre, Wageningen, The Netherlands; Virginia Tech, United States of America

## Abstract

The daisy-like flowers of pyrethrum (*Tanacetum cinerariifolium*) are used to extract pyrethrins, a botanical insecticide with a long history of safe and effective use. Pyrethrum flowers also contain other potential defense compounds, particularly sesquiterpene lactones (STLs), which represent problematic allergenic residues in the extracts that are removed by the pyrethrum industry. The STLs are stored in glandular trichomes present on the pyrethrum achenes, and have been shown to be active against herbivores, micro-organisms and in the below-ground competition with other plants. Despite these reported bioactivities and industrial significance, the biosynthetic origin of pyrethrum sesquiterpene lactones remains unknown. In the present study, we show that germacratrien-12-oic acid is most likely the central precursor for all sesquiterpene lactones present in pyrethrum. The formation of the lactone ring depends on the regio- (C6 or C8) and stereo-selective (α or β) hydroxylation of germacratrien-12-oic acid. Candidate genes implicated in three committed steps leading from farnesyl diphosphate to STL and other oxygenated derivatives of germacratrien-12-oic acid were retrieved from a pyrethrum trichome EST library, cloned, and characterized in yeast and *in planta*. The diversity and distribution of sesquiterpene lactones in different tissues and the correlation with the expression of these genes are shown and discussed.

## Introduction

Pyrethrum has a long history of cultivation to produce insecticides based on pyrethrins that are extracted from the flower heads. The flower heads consist of a collection of small flowers: ray and disc florets that are set on a receptacle. Microscopic examination of the disc florets shows that the surface of the achenes is densely covered by glandular trichomes. In our previous work, we showed that glandular trichomes covering pyrethrum achenes and leaves are filled with a mixture of compounds dominated by sesquiterpene lactones (STLs) [1]. In earlier publications these STLs were characterized in more detail [2]–[6]. Pyrethrosin, the first recognized STL isolated from the flower heads of *Tanacetum cinerariifolium,* exhibits several biological properties including cytotoxic [7], phytotoxic [8], antibacterial [9], antifungal [10] and root growth inhibitory [4] activities. Before, we also showed that the trichome content - with STLs as the major constituent - has antifeedant activity against herbivores and is fungistatic against seedling-specific pyrethrum pathogens, suggesting a maternal protection mechanism promoting survival of the next generation [1]. Apart from these reported bioactivities, pyrethrum STLs were shown to be responsible for allergic reactions reported for pyrethrum extracts [11]–[13]. Considerable efforts have since resulted in refining procedures yielding pyrethrin oil preparations containing only trace amounts of STLs which no longer cause dermatitis.

**Figure 1:**
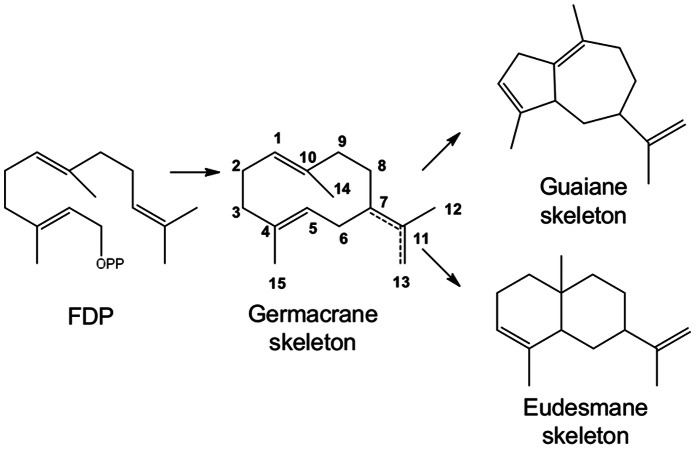
Simplified scheme for the formation of germacrane, eudesmane and guaiane-type sesquiterpene lactones. The germacrane skeleton is formed from farnesyl diphosphate (FDP) and undergoes a cyclization reaction to either the guaiane or the eudesmane skeleton [43].

**Figure 2:**
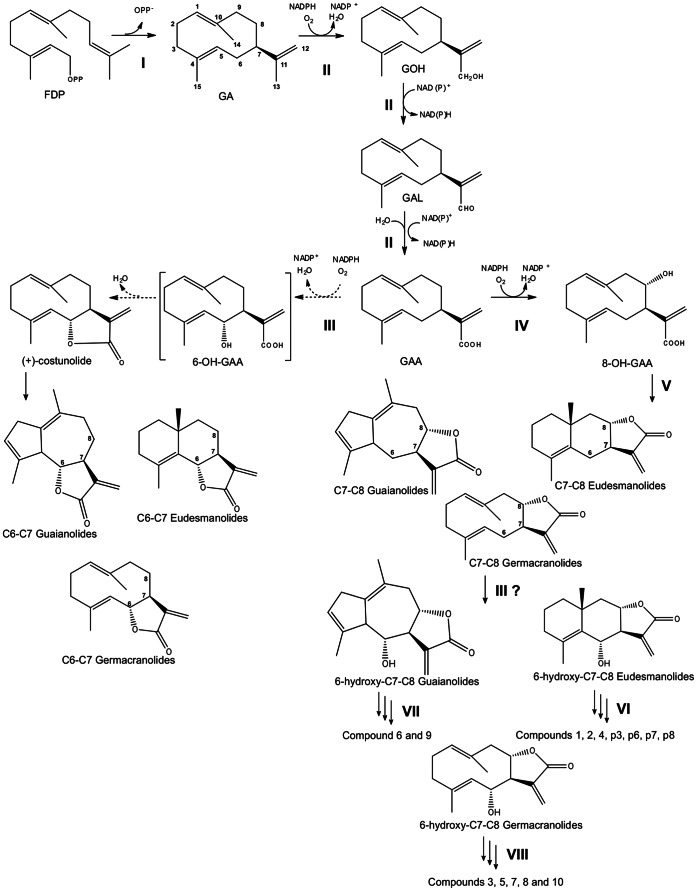
Proposed biosynthetic routes to C6–C7 and C7–C8 sesquiterpene lactones. I, Cyclization of farnesyl diphosphate (FPP) to (+)− germacrene A (GA) by (+)− germacrene A synthase (*TcGAS*). II, Oxidation of GA via germacra-1(10),4,11(13)-trien-12-ol (GOH) and germacra-1(10),4,11(13)-trien-12-al (GAL) into germacra-1(10),4,11(13)-trien-12-oic acid (GAA) catalyzed by an NADPH-dependent single P450 enzyme (*TcGAO*). III, Hydroxylation at the C6 position of GAA or of various C7–C8 lactones by a second P450 enzyme (*TcCOS*). IV, Hydroxylation at the C8 position of GAA, and V, subsequent spontaneous lactonization. VI, VII, and VII involve extra oxidative steps and esterification with glycosyl, tigloyl and acyl groups, and cyclization of the sesquiterpene backbone to get to the structures in Figure 3 and 4.

Both the potentially interesting bioactivities of STLs as well as the refinery costs they present to the industry raised our interest to elucidate the mechanism by which these compounds are produced. Even though the detailed structures of STLs vary across the Asteraceae family, their basic structure consists of a C15 sesquiterpene backbone and a lactone moiety. The sesquiterpene backbones are mainly germacranolide, eudesmanolide, and guaianolide skeletons [14], [15], which have all been suggested to be derived from germacrene A (Figure 1) [16]. In addition to their sesquiterpene backbone, the regio- (C_6_ or C_8_) and stereoselective (α or β) formation of the lactone ring also contributes to the diversity in STLs reported in nature. The proposed pathway to the STLs starts with the cyclization of farnesyl diphosphate (FDP) to germacrene A by germacrene A synthase (GAS). In the next step, germacrene A is oxidized at its isopropenyl side chain by a single cytochrome P450 enzyme, germacrene A oxidase (GAO), to form germacra-1(10),4,11(13)-trien-12-ol (GOL), which is then further oxidized to germacra-1(10),4,11(13)-trien-12-al (GAL) and germacra-1(10),4,11(13)-trien-12-oic acid (GAA) [17] (Figure 2). Hydroxylation of GAA at the C6-α position by costunolide synthase (COS) results in an unstable intermediate, 6α-OH-GAA, which cyclises to costunolide, the precursor of the C6–C7 costunolide-types of sesquiterpene lactones (C6–C7 STLs) [18], [19]. Hydroxylation of GAA at the C8-β position, in a reaction that also depends on a cytochrome P450 enzyme [20], results in 8β-OH-GAA. Computational modeling showed that the atomic distance between the hydroxyl oxygen and the carbonyl carbon that forms the C-O bond in the lactonization is 0.87 Å longer in 8β-OH-GAA than in 6α-OH-GAA. This longer atomic distance excludes spontaneous lactonization, suggesting that an enzyme must be involved in the formation of the C7–C8 *cis*-type sesquiterpene lactones as occurring in sunflower (C7–C8 *cis* STLs) [20], [21].

**Figure 3:**
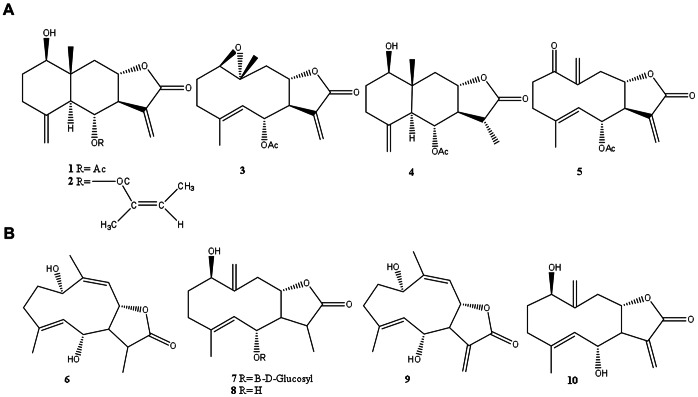
Major sesquiterpene lactones reported for pyrethrum. A, β-cyclopyrethrosin (1), chrysanin (2), pyrethrosin (3), dehydro-β-cyclopyrethrosin (4), chrysanolide (5) [3]. B, (11R)-11,13-dehydro-tatridin-A (6), (11-R)-6-O-β-D-glucosyl-11–13 dehydro-tatridin-B (7), (11R)-11,13-dehydro-tatridin-B (8) tatridin-A (9), and tatridin-B (10) [4].

All known sesquiterpene lactones from pyrethrum are C7–C8 type STLs with a *trans* conformation [3]–[5] and are all likely formed from 8α-OH-GAA, which is expected to lactonize spontaneously just as 6α-OH-GAA (Figure 2). Interestingly, all are also hydroxylated at the C6-α position (Figure 3). For these reasons we hypothesized that the three enzymes catalyzing the formation of costunolide from FDP in Asteraceae relatives of pyrethrum, are present in pyrethrum and catalyze part of the formation of the pyrethrum 6α-hydroxylated C7–C8 *trans* STLs found in pyrethrum trichomes. To identify the corresponding genes, trichomes of pyrethrum were isolated and used to generate an EST contig library from which gene candidates were retrieved, cloned and characterized in yeast and *in planta*. Transcriptional profiles in specific tissues and along flower development were correlated with chemical profiles of the reaction products to confirm the involvement of these genes in the production of pyrethrum sesquiterpene lactones.

## Results

### Localization of Sesquiterpene Lactones in Pyrethrum

**Figure 4:**
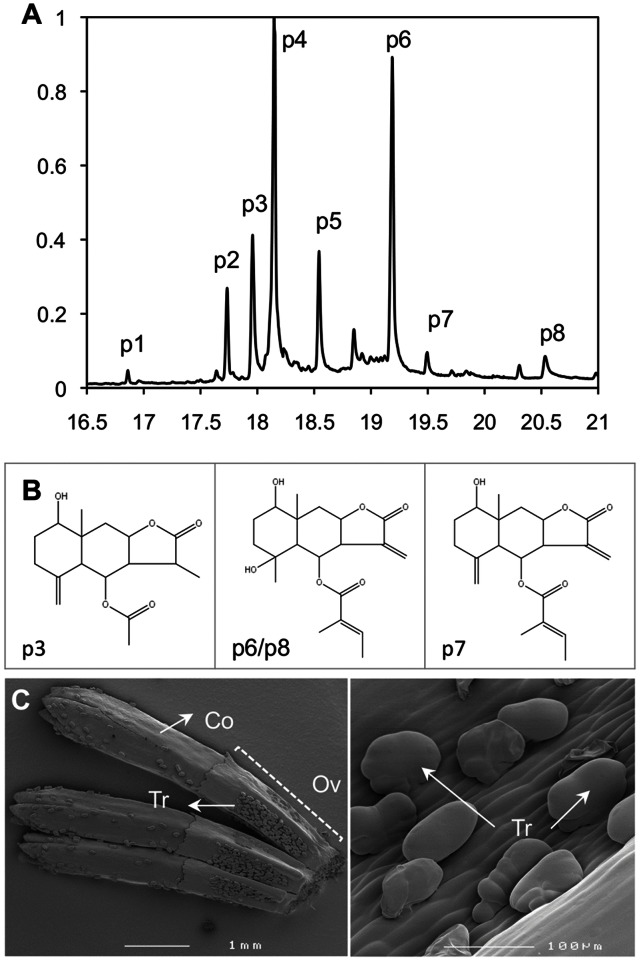
Glandular trichome content of pyrethrum achenes. A, GC-MS analysis of chloroform dips of seed extracts, representing the content of trichomes. B, Putative chemical structures of the STLs found in pyrethrum trichomes according to a NIST library search. 1.8-hydroxy-3, 8a-dimethyl-5-methylene-2-oxododecahydronaphthol(2,3-b)furan-4yl acetate (p3), 1.8-hydroxy-8a-methyl-3,5-dimethylene-2-oxododecahydronaphthol(2,3-b)furan-4yl(2E)-2-methyl-2-butenoate (p7), and 2-methylbut-2enoic acid (5,8-dihydroxy-5,8a-dimethyl-3-methylene-2-oxo-dodecahydronaphtho(2,3-b)furan-4-yl) ester (p6/p8). The prefix “p” stands for putative. Compound p2 is probably an oxygenated sesquiterpene, p4 and p5 are likely to be STLs of undetermined structure, and p1 is an unknown compound. Table S1 provides further details. C, cryo-scanning electron microscopy image of complete, unopened disk florets showing the highest density of glandular trichomes in the indentations between the ribs of the ovaries (left panel) and closer view of trichomes (right panel). Ov, ovary; Co, corolla; Tr, trichome.

In our previous study we showed that sesquiterpene lactones (STLs) accumulate in glandular trichomes of achenes and leaves [1]. To establish the localization of sesquiterpene lactones in other tissues as well, we analyzed by GC-MS, dichloromethane (DCM) extracts of (i) leaves, (ii) stems, (iii) disk florets and (iv) ray florets. We tried to assign the most significant peaks in the chromatogram (Figure 4) by comparison of their mass spectrum with the NIST library followed by visual inspection (Table S1). This resulted in the putative identification of four eudesmane C7–C8 STLs (peaks 3, 6, 7, 8) and two STLs of unknown structure (peak 4 and 5). Peak 2 is likely an oxygenated sesquiterpene and peak 1 could not be identified.

**Figure 5:**
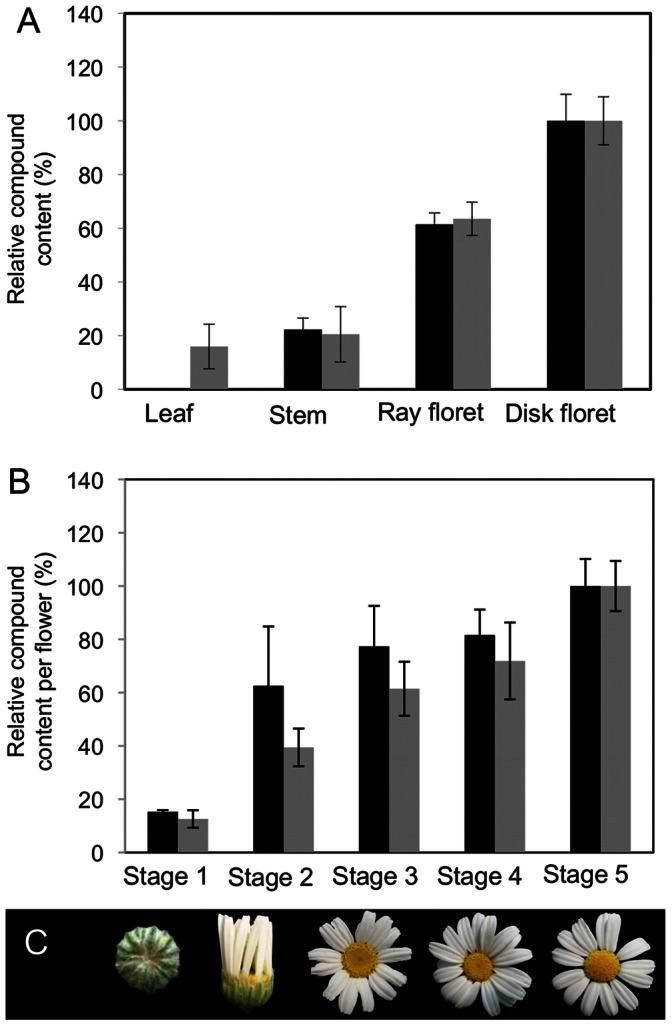
Pyrethrum sesquiterpene lactones content in different tissues. Peak areas of a C7–C8 type sesquiterpene lactone (Grey bars: compound p7 of Figure 4), and of a STL of undetermined structure Black bars: compound p5 of Figure 4) in pyrethrum leaves, stems, ray florets and disk florets relative to the peak areas in disk florets (100%) (A); and in pyrethrum flowers in different developmental stages relative to the peak areas in stage 5 flowers (B). Images of the corresponding pyrethrum flower heads (C) Error bars represent SEM (N = 3).

Comparison of the relative peak areas of one C7–C8 STL (Figure 4, peak 7) and one unidentified STL (Figure 4, peak 5) showed that the highest concentrations were found in the disk and ray florets, while leaves and stems displayed lower concentrations (Figure 5A). Both lactones were present in all studied tissues except leaves, in which only the C7–C8 STL was not detected.

To determine how STLs accumulate during flower development, extracts of flower heads in different developmental stages (Figure 5C) were analyzed for STL content. For both STLs analyzed (Figure 4 peak 7 and peak 5), the concentration per flower increased gradually from stage 1 to stage 5 (Figure 5B).

### Isolation of Putative STL Biosynthesis Related Genes

Young leaves, ovaries isolated from stage 3 flowers, and trichomes isolated from ovaries of stage 4 and 5 flowers were used to generate cDNA libraries. 454 Sequencing gave 484.392 reads of an average size of 400 bp, which after assembly resulted in 27.317 contigs and 144.825 reads that remained as singletons. Although 85% of the contigs had an average size of 400 bp, a small percentage of them (1.2%) had a size over 1200 bp, which represents the average size of a full-length gene. Sequences were annotated by blasting against GenBank (http://blast.ncbi.nlm.nih.gov).

**Figure 6:**
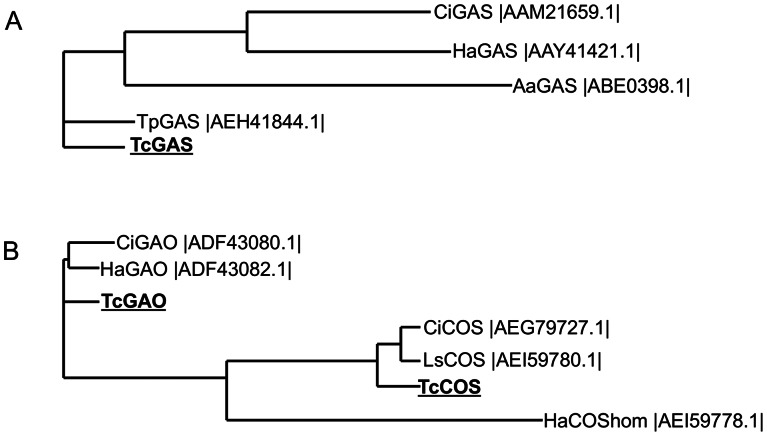
Phylogenetic analyses of the germacrene A synthases and oxidases. A, tree based on the deduced amino acid sequences of the putative pyrethrum germacrene A synthase (*TcGAS*) and other characterized plant *GAS* genes. B, tree based on the putative pyrethrum germacrene A oxidase (*TcGAO*) and costunolide synthase (*TcCOS*) and other characterized plant GAO and COS genes. The tree was constructed by Neighbour-Joining method using ClustalW2 (http://www.ebi.ac.uk/Tools/msa/clustalw2). The species abbreviations are *Ci, Cichorium intybus; Ha, Helianthus annuus; Ls, Lactuca sativa; Tp, Tanacetum parthenium; Tc, Tanacetum cinerariifolium.*

The genes responsible for C6–C7 *trans* STL biosynthesis in *Tanacetum parthenium* (feverfew) (germacrene A synthase, *TpGAS*, AEH41844.1) [22], and *Cichorium intybus,* (germacrene A oxidase, *CiGAO*, ADF43080.1 [23], and costunolide synthase, *CiCOS*, AEG79727.1) [19], and for C7–C8 *cis* STL biosynthesis in *Helianthus annuus* (sunflower) (8β-OH-GAA synthase, *HaG8H*, AEI59778.1) [20] were blasted against the pyrethrum trichome-specific EST contig library. This resulted in the identification of three highly homologous full-length ESTs from pyrethrum. However, the EST trichome database did not have ESTs highly homologous to *HaG8H*, and the most homologous EST found was short and displayed an identity of less than 45%. The first highly homologous sequence, *TcGAS* (KC441526) encoded a protein with 95% similarity to the feverfew germacrene A synthase (AEH41844.1) and 83 to 86% similarity to the germacrene A synthases from *Artemisia annua* (ABE0398.1), *H. annuus* (AAY41421.1) and *C. intybus* (AAM21659.1) (Figure 6A). The second sequence, *TcGAO* (KC441527) encoded a protein with 88% similarity to the *C. intybus* (ADF43080.1) and the *H. annuus* (ADF43082.1) germacrene A oxidases, and the third one (*TcCOS*) (KC441528) showed 89% similarity to the *C. intybus* (AEG79727.1) and *Lactuca sativa* (AEI59780.1) costunolide synthases, sharing only 32% identity with *HaG8H* (Figure 6B and Figures S1, S2, S3).

### Functional Characterization of Pyrethrum GAS/GAO/COS in Yeast and *in planta*

**Figure 7:**
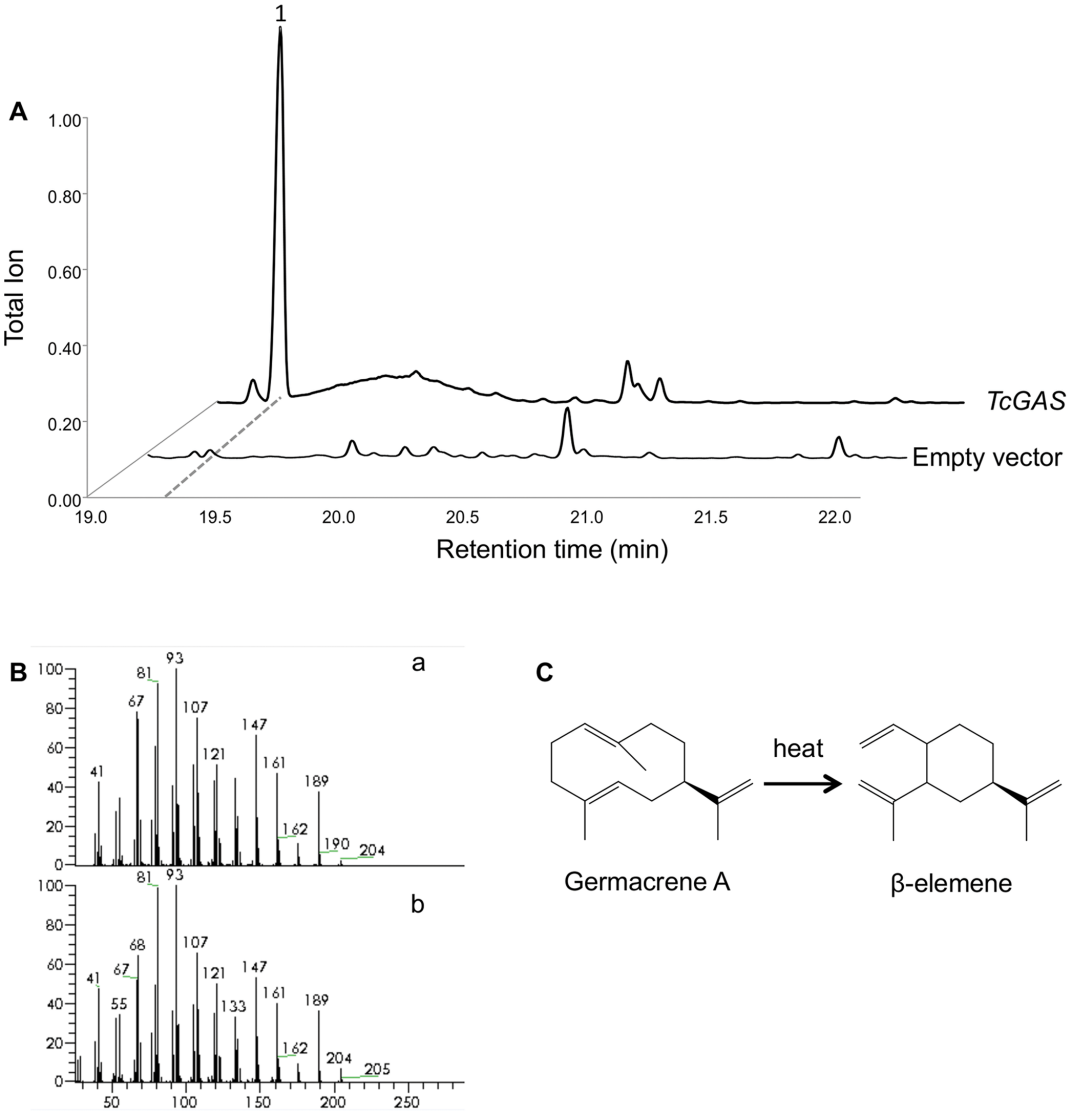
Germacrene A production *in planta*. A, headspace analysis of volatiles emitted from *Nicotiana benthamiana* leaves agro-infiltrated with *TcGAS*. B, mass fragmentation pattern of compound 1 (Ba) and β-elemene from the Wiley library (Bb). C, cope rearrangement of germacrene A to β-elemene by heat.

The full length cDNA of the putative germacrene A synthase identified in pyrethrum, *TcGAS*, was cloned into a binary expression vector under the control of the Rubisco small subunit promoter and introduced into *A. tumefaciens* for *in planta* expression. *Nicotiana benthamiana* leaves were agro-infiltrated and analyzed after 5 days according to van Herpen *et al*. [24]. In the headspace of *TcGAS* agro-infiltrated *N. benthamiana* leaves we detected a peak, which was not present in the headspace of empty vector infiltrated leaves (Figure 7A), and for which the mass spectrum matched the mass spectrum of β-elemene (Figure 7B), the on-column, heat-induced Cope rearrangement product of germacrene A (Figure 7C) [14]. Expression in yeast gave similar results confirming the product specificity of TcGAS (data not shown).

**Figure 8:**
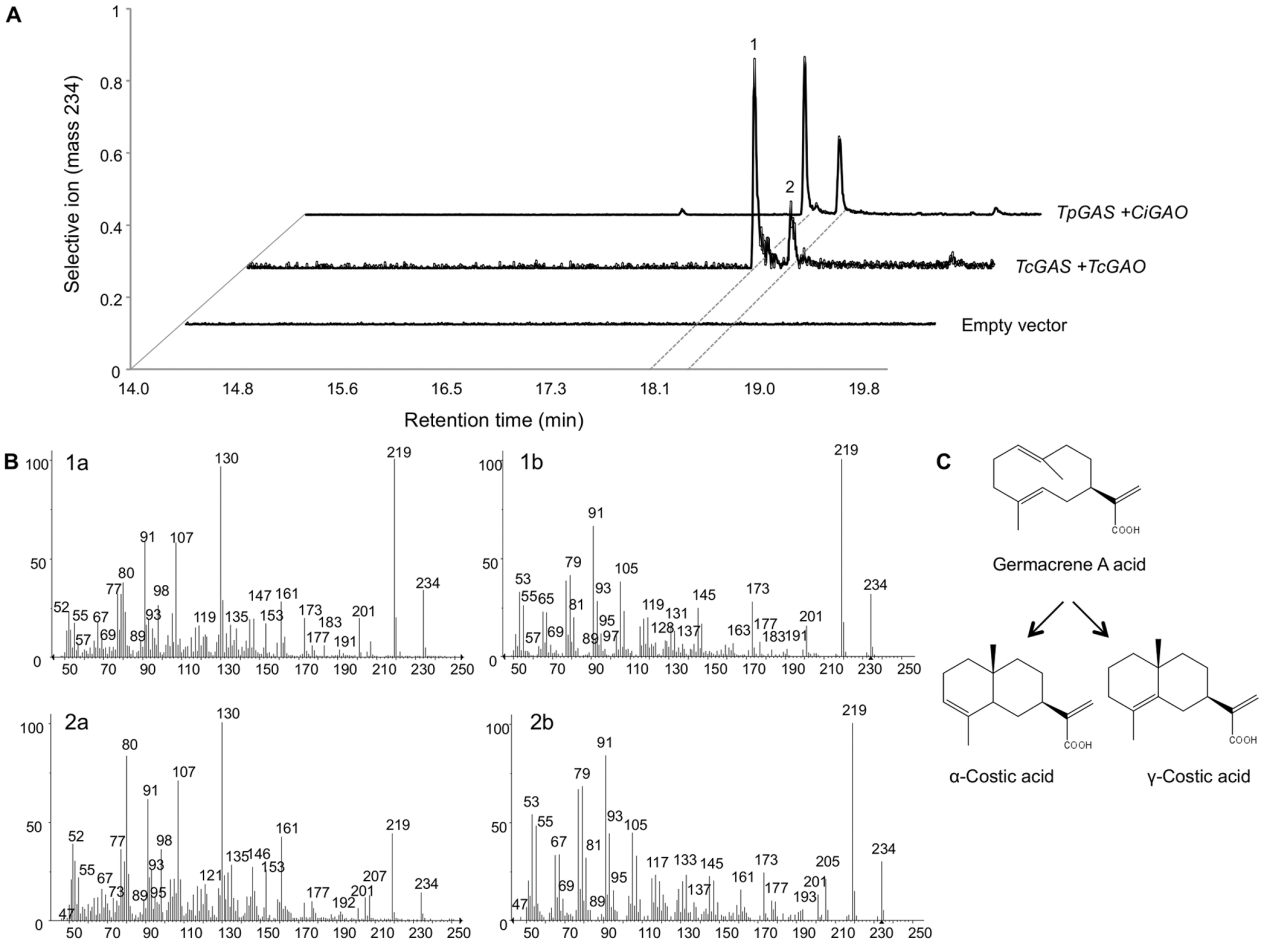
α-costic acid and γ-costic acid production in yeast. A, GC-MS chromatograms for the terpenoids with a parent mass of m/s 234 isolated from yeast transformed with the indicated genes. Front line showing the metabolites for the empty vector control, middle line showing the metabolites of yeast transformed with *TcGAS* and *TcGAO*, and last line showing the metabolites of yeast transformed with *Tanacetum parthenium TpGAS* and *Cichorium intybus CiGAO*. B, mass fragmentation pattern of peak 1 (1a), α-costic acid (1b), peak 2 (2a), and γ-costic acid (2b). C, acid induced rearrangement of germacrene A acid to α-costic acid and γ-costic acid.

To assess the enzymatic activity of *TcGAO*, its open reading frame (ORF) and the ORF of *TcGAS* were expressed using the yeast dual expression vector pESC-TRP under the control of GAL10 and GAL1 promoters. This plasmid (*TcGAS*+*TcGAO*::pESC-TRP) and the previously characterized feverfew *GAS* and chicory *GAO*, *TpGAS*+*CiGAO*::pESC-TRP [19], were transformed into the yeast strain WAT11. After induction of expression of the two genes, metabolites were extracted and analyzed using GC-MS. Two compounds that were not present in yeast transformed with the empty vector were detected in yeast expressing pyrethrum *TcGAS+TcGAO* as well as *TpGAS+CiGAO* (Figure 8A). The mass fragmentation (Figure 8B) patterns of the two peaks matched the fragmentation patterns of the cyclization products of GAA under acidic conditions, γ-costic acid and β-costic acid (Figure 8C) [17], [23]. Considering that the earlier published *CiGAO* [19] is highly similar and yielded the exact same costic acid products as *TcGAO*, the encoded enzyme was designated as germacrene A oxidase (TcGAO) (CYP71AV2).

**Figure 9:**
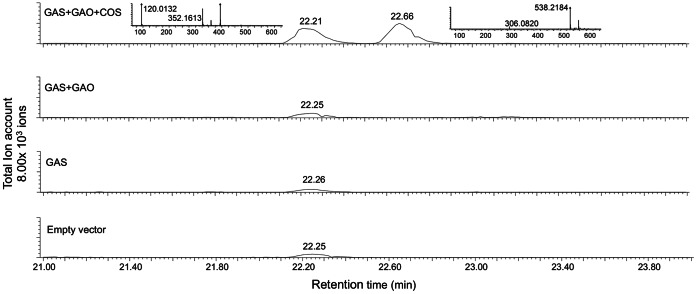
Costunolide production *in planta*. LC-QTOF-MS analysis of non-volatiles metabolites from *Nicotiana benthamiana* leaves agro-infiltrated with empty vector, *TcGAS*, *TcGAS*+*TcGAO*, and *TcGAS+TcGAO+TcCOS*. The MS spectrum of the two new peaks (peak 22.21 and peak 22.66) in leaves agro-infiltrated with pyrethrum GAS+GAO+COS and their respective parent ions ([m/z]^−^ 352.1613 and 538.2184, respectively) are shown.

The full-length cDNA of the costunolide synthase candidate, *TcCO*S, was cloned into a yeast expression vector and co-transformed with *TcGAS* and *CiGAO* into yeast. The transformed yeast culture was able to produce costunolide. However, only a very small amount was detected by GC-MS, which was confirmed by a parallel injection of a commercially available standard (data not shown), indicating that *TcCOS* encodes a costunolide synthase (TcCOS) (CYP71BL4). Low costunolide production by COS has been shown to be due to the increasingly acidic conditions during yeast culturing, which can be improved by buffering [23]. During our *in-vitro* yeast assay we took care of this aspect, and, therefore, we presume that the relatively poor efficiency may be due to the fact that *TcCOS* prefers C7–C8 lactonized substrates rather than GAA. Nevertheless, apparently TcCOS has 6α-hydroxylase activity which results in 6α-OH-GAA that spontaneously lactonizes to costunolide which can then be precursor for the C6–C7 costunolide type STLs not yet reported in pyrethrum. To confirm the activity of this putative pyrethrum *TcCOS in planta*, cDNAs of *TcGAS*, *TcGAO* and *TcCOS* were cloned into a binary vector under control of the Rubisco promoter, and introduced into *A. tumefaciens*. *A. tumefaciens* cultures with RBC::*TcGAS*, RBC::*TcGAO* and RBC::*TcCOS* were co-infiltrated in *N. benthamiana* leaves. After 5 days, methanol extracts were prepared and analysed using LC-QTOF-MS. Comparison of chromatograms showed two new compounds, eluting at 22.21 and 22.66 min, in leaves infiltrated with *TcGAS+TcGAO+TcCOS*, which were not present in leaves infiltrated with *TcGAS+TcGAO*, *TcGAS* alone, or empty vector control (Figure 9). The parent masses of these two new peaks, 352.1609 (at 22.21) and 538.2184 (at 22.66) were within 6 and 22 ppm from the elemental formulas of costunolide-cysteine (C_3_H_7_NO_2_S) and costunolide-glutathione (C_10_H_17_N_3_O_6_S) conjugates, respectively. Such costunolide conjugates were also previously identified in a similar study where *N. benthamiana* leaves were agro-infiltrated with the chicory orthologs of these genes [19].

### Expression Analysis of Pyrethrum STL-biosynthesis Related Genes

**Figure 10:**
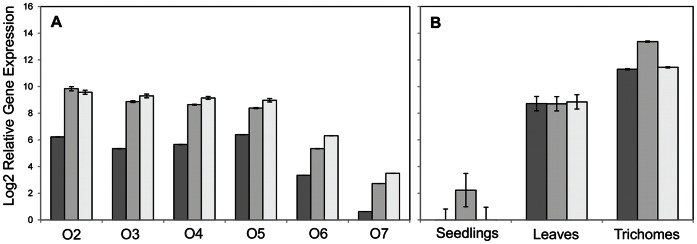
Log_2_ of the Relative Gene Expression of pyrethrum *TcGAS*, *TcGAO* and *TcCOS* in different tissues. A, Expression in ovaries isolated from flowers at different developmental stages (O2 to O7). B, expression in seedlings, leaves and trichomes isolated from stage 3 ovaries. Black bars represent *TcGAS*, grey bars represent *TcGAO* and white bars represent *TcCOS*. The Ct value for each sample was normalized using *GAPDH* as housekeeping gene. Error bars represent SEM (N = 3).

In order to assess the expression of *TcGA*S, *TcGAO* and *TcCOS*, and to confirm that the expression is exclusively in trichomes, RT-qPCR experiments were carried out using cDNA samples from seedlings (without trichomes), young leaves, and trichomes alone (isolated from achenes of stage 3 flowers). Comparison of the relative gene expression (RGE) of the three genes revealed that expression was highest in the trichomes, lower in the leaves, and absent in seedlings (Figure 10A). RT-qPCR analysis of ovaries derived from flowers in different stages of development revealed a similar pattern of expression for all three genes (Figure 10B). Expression was high and almost constant in stages 2, 3, 4, and 5 and gradually decreased in stages 6 and 7.

## Discussion

In the present study we demonstrate in yeast and *in planta* that the three pyrethrum genes, *TcGAS*, *TcGAO* and *TcCOS* encode enzymes, which catalyze the formation of costunolide from FDP, through the unstable intermediate 6α-OH-GAA. The C6–C7 costunolide-like sesquiterpene lactones are important constituents of the Asteraceae as they have a range of biological activities [25]–[28]. Costunolide formation, however, is likely not the major function of TcCOS as the 6α-hydroxylation is also crucial in the biosynthesis of the major class of pyrethrum STLs, the C7–C8 STLs, which are usually also oxidized at C6 position with α stereochemistry. We were, however, unable to identify the putative cytochrome P450 responsible for C7–C8 lactone ring formation in pyrethrum.

### Accumulation of Different Types of STLs in Trichomes

Previous studies in pyrethrum and other species of the Asteraceae family have shown that sesquiterpene lactones (STLs) are exclusively stored in trichomes and probably also produced there [1], [22], [29]–[31]. Here, we investigated this for pyrethrum and observed that the concentrations of two STLs, a C7–C8 (p7) type and an STL of unknown structure (p5) in various tissues, indeed correlated with the presence of trichomes (Figure 5A) [1]. Quantitative expression analysis on isolated trichomes confirmed that all three genes putatively involved in pyrethrum STL biosynthesis are highly expressed in trichomes, and share a highly similar expression pattern (Figure 10B). Consequently, based on the results presented here and on what has been reported for feverfew, a close relative of pyrethrum [22], glandular trichomes are the sites of sesquiterpene lactone accumulation and production also in pyrethrum. STL accumulation per flower increased gradually in flower development stages 1 to 5 and was accompanied by a high and constant expression of the three genes involved in their production, as long as disc florets were still opening (Figure 5B, 10A). When all florets had opened gene expression was down regulated. Consistent with other reports on terpene biosynthesis [32]–[34], and with our previous report on pyrethrin accumulation [1], the results reported here indicate that also STL accumulation is developmentally regulated in pyrethrum. Even though both STLs and pyrethrins accumulated in a similar pattern during flower development, and both were entirely (STLs) or partially (pyrethrins) produced by the same glandular trichomes, the final concentrations of STLs are 5–10 fold lower than of pyrethrins. These differences might represent ecologically optimal adaptations to specific herbivores and pathogens or surrounding competing flora [1], [4] at the lowest fitness cost [1].

### Role of C6-α Hydroxylation of GAA in the Biosynthesis of Pyrethrum STLs

All pyrethrum STLs reported in the literature so far belong to the C7–C8 *trans* type (Figure 3), rather than to the C6–C7 *trans* type typical of costunolide-like STLs [3]–[5]. Yet, their basic sesquiterpene backbone, before lactone ring formation, is likely to be derived from the same germacratrien-12-oic acid (GAA) precursor, and, hence, similar enzymes are expected to be involved. The formation of the reported pyrethrum STLs [3]–[5], seems to depend on C8-α hydroxylation of GAA, but a gene for that step has not been described yet in any species. C8 hydroxylation of GAA in β-position on the other hand is known to be catalyzed by *HaG8H* from sunflower. Pyrethrum did not contain a homologue of *HaG8H*. This could imply that the stereospecific 8α-hydroxylation requires quite a different enzyme. Interestingly, however, all pyrethrum C7–C8 STLs also display hydroxylation at the C6-α position (Figure 3). As both C6-α and C8-α hydroxylated GAA would spontaneously and irreversibly form a lactone ring to form either C6–C7 or C7–C8 *trans* STLs [20], it seems most likely that 6α-hydroxylation by TcCOS *in planta* occurs after a yet unknown enzymatic 8α-hydroxylation and spontaneous formation of the C7–C8 lactone ring. Figure 2 shows how we envisage the precursors of the reported STLs to be formed. We presume that the relatively poor efficiency of TcCOS on GAA may be due to the fact that it prefers a C7–C8 lactonized substrate rather than GAA. Rapid C6-hydroxylation of GAA would after all inevitably lead to the formation of C6–C7 types of STLs, which have not been reported in pyrethrum.

Additional enzymatic reactions would require other P450 enzymes, for example catalyzing hydroxylation at C1 (Figure 3, all compounds; Figure 4, compounds p3, p7, p6/8) or epoxidation of a C1–C2 double bond (Figure 3, compound 3). The hydroxyl group could be subsequently oxidized to a ketone (Figure 3, compound 5) catalyzed by the same or a second P450 enzyme or a dehydrogenase. In some compounds the C3 and C6 hydroxyl groups are further acetylated (Figure 3, compounds 1, 3, 4, 5; Figure 4, compound p3), glycosylated (Figure 3, compound 7), or tigloylated (Figure 3 compounds 2; Figure 4 compounds p7, p6 and p8), by presumably acetyl, glycosyl and acyl transferases, respectively. Finally germacrene cyclases would be necessary to catalyze the cyclization of the sesquiterpene backbone (Figure 3, compounds 1, 2 and 4; Figure 4 compounds p3, p7, p6 and p8). Out of the 27.317 ESTs, the pyrethrum EST database contains, 155 ESTs with homology to acyl transferases, between 100 and 250 ESTs that match to acetyl and glycosyl transferases respectively, 30 ESTs matching cyclases and more than 150 matching cytochrome P450s. In consequence, in the absence of close homologues that would facilitate the selection of the enzymes required to catalyze the missing steps in the pyrethrum STLs biosynthesis, other strategies, like similarity in the pattern and level of expression with the genes already characterized in the present study, would have to be applied to narrow down these candidate numbers.

The lack of proper standards, the lack of verified GC-MS spectra and KI values for compounds reported in the literature and the glycosylation of some of the reported pyrethrum STLs, resulted in poor identifications for most pyrethrum STLs that we here report. Only compounds p3, p6, p7 and p8 were satisfactorily identified (Figure 3, and Table S1).

In summary, we have isolated and characterized two genes (*TcGAS* and *TcGAO*) involved in the biosynthesis of the pyrethrum sesquiterpene lactone backbone, GAA, which is the central precursor of all known STLs found in pyrethrum. Furthermore, a gene encoding an enzyme capable of catalyzing the 6α-hydroxylation of GAA was characterized. In heterologous expression, this hydroxylation yields costunolide, however, the enzyme possibly also (or preferably) catalyzes hydroxylation at the C6 position of the – likely already lactonized - precursor of the reported C7–C8 *trans* type STLs in pyrethrum. To proof this hypothesis the pyrethrum 8α-hydroxylase of GAA would need to be identified.

## Materials and Methods

### Plant Material

Seeds were obtained from Honghe Senju Biology Co. Ltd. (Kunming, Yunnan, China).

*T. cinerariifolium* plants were grown in open field conditions at PRI with irrigation and fertilization when needed.

### Extraction and Analysis of Sesquiterpene Lactone Contents

Plant materials, including leaves, stems, seedlings, ray florets, disk florets, receptacle and flowers of five developmental stages (1 to 5) as defined by Head (1966) [35] were dissected from flowers picked during summer, flash frozen in liquid nitrogen and ground to a fine powder. Apolar metabolites were extracted from these tissues (50 mg) by 30 sec of vortexing and 5 min sonication in 1 ml chloroform. The extracts were centrifuged for 5 min at 3500 rpm, dehydrated using anhydrous Na_2_SO_4_ and analyzed by gas chromatography-mass spectrometry (GC-MS). Ovary secretory trichomes, ovaries without trichomes and intact ovaries were isolated from stage 3 flowers and extracted as previously described [1].

### GC-MS Analysis of Plant Extracts

The GC-MS measurements were conducted on an Agilent 7890A gas chromatograph consisting of a 7683 series autosampler, 7683B series injector, and 5975C inert MSD with triple-axis detector. Control of the equipment, data acquisition, processing, and management of chromatographic information were performed using the Agilent Enhanced ChemStation E.02.00.493 software. A Zebron ZB-5MS GC13 capillary column (30 m×0.25 mm i.d. ×0.25 µm film thickness; Phenomenex, USA) with 5 m guard column was employed for the chromatographic analyses, which were based on an established pyrethrin protocol. The injector temperature of the GC was set at 250°C and helium was the carrier gas with a column flow rate of 1.0 ml/min. The injection volume was 1 µl and samples were injected in splitless mode. The oven temperature was held at 45°C for 2 min and programmed to 300°C at 15°C/min, the final temperature was held for 4 min. Total run time per sample was 23 min. The mass spectrometer was operated in the electron ionisation mode (70 eV) with an ion source temperature of 230°C. The detector was switched on after 4.5 min solvent delay and the full mass-range mode was used for the analyses of the samples with a mass to charge ratio range (m/z) from 45–250 atomic mass units (amu), and a scan time of 0.2 sec and an inter-scan delay of 0.1 sec. If not described otherwise, samples were prepared in CHCl_3_ and diluted 5× before injection. Constituents of the essential oil were identified by comparing their mass spectra with those of the reference library, the NIST 08 mass spectral database. Putative identification was based on similarity calculated by the NIST library programme followed by visual inspection.

### Isolation and Amplification of Putative STL Biosynthesis Related Genes

An expressed sequence tag (EST) database of three cDNA libraries derived from pyrethrum leaves, ovaries and trichomes was produced using the GS FLX Titanium platform. Reads were clustered and assembled into contigs. Using an in-house bioinformatics facility, potential gene functions of the resulting contigs were identified by blasting against the Nr database of annotated genes and storing the first 50 hits in a local database. The three candidate STL biosynthesis related contigs were identified by sequence homology to known sesquiterpene synthases and P450s. The candidate genes were amplified from trichome cDNA using high fidelity Phusion polymerase (Finnzymes), cloned into pGEMT-easy vector (Promega), and sequenced. The cDNA sequences for the pyrethrum germacrene A synthase (*TcGAS*), the germacrene A oxidase (*TcGAO*) and the costunolide synthase (*TcCOS*) have been deposited in GenBank under the accession numbers KC441526, KC441527, and KC441528, respectively. The sequences for *TcGAO* and *TcCOS* were also submitted to David Nelson’s cytochrome P450 homepage (http://drnelson.uthsc.edu/cytochromeP450.html) and were assigned the names CYP71AV2 and CYP71BL4, respectively [36]. Access to the database is available based on a Material Transfer Agreement obtainable through the corresponding author.

### Co-expression of *TcGAO* and *TcCOS* with *TcGAS* in Yeast

For the production of GAA in yeast, *TcGAO* and *TcGAS* were both cloned into the pESC-Trp yeast expression vector (Agilent technologies) with the TRP1 auxotrophic selection marker. *TcGAS* was amplified from trichomes cDNA using high fidelity Phusion polymerase (Finnzymes) with the addition of *Not*I/*Bgl*I restriction sites. The amplified product was digested by *Not*I/*Bgl*I and ligated into the pESC-Trp plasmid. Subsequently, *TcGAO* was amplified and cloned into *TcGAS* pESC-Trp using *Sal*I/*Kpn*I restriction sites, yielding the final plasmid *TcGAS*+*CiGAO* pESC-Trp. Finally, *TcCOS* was cloned into modified pYEDP60k [37] using *Not*I/*Pac*I restriction sites.

The *TcGAS*+*CiGAO* pESC-Trp plasmid was transformed into the WAT11 [38] yeast strain and the clones were selected on synthetic dextrose (SD) minimal medium (0.67% Difco yeast nitrogen base medium without amino acids, 2% D-glucose, 2% agar) supplemented with amino acids, but omitting L-tryptophane for auxotrophic selection of transformants.

*TcGAS*+*CiGAO* pESC-Trp and pYEDP60k plasmids containing *TcCOS* were co-transformed into the WAT11 yeast strain. After transformation yeast clones containing both plasmids were selected on SD minimal medium supplemented with amino acids, but omitting uracil, adenine sulphate and L-tryptophane for auxotrophic selection of transformants.

For the induction of gene expression in yeast, the transformed WAT11 yeast strain with *TcGAS*+*CiGAO* pESC-Trp or co-transformed with *TcGAS*+*CiGAO* pESC-Trp and *TcCOS* PYEDP60k-Ura-Ade were inoculated in 3 mL SD minimal medium (0.67% Difco yeast nitrogen base medium without amino acids, 2% D-dextrose) but omitting Trp or Trp-Ura-Ade amino acids, respectively. The yeast was cultured overnight at 30°C and 300 rpm. The start culture was diluted to OD 0.05 in SG (0.67% Difco yeast nitrogen base medium without amino acids, 2% D-galactose) minimal medium omitting Trp or Trp-Ura-Ade amino acids, respectively. All yeast induction experiments were performed in triplicates in 50 mL of culture volume. Cultures were buffered at pH 7.5 using 75 mM HEPES. After fermentation for 48 h at 30°C and 300 rpm, the medium was extracted with 20 mL ethyl acetate. From this, a 10 mL sample was taken and the ethyl acetate evaporated with a stream of N_2_ to a final volume of 1 mL, which was analyzed by GC-MS.

### Plasmid Construction for Expression in *Nicotiana benthamiana*

For expression in *N. benthamiana*, *TcGAS*, *TcGAO* and *TcCOS* were cloned into ImpactVector1.1 (http://www.impactvector.com/) to express them under the control of the Rubisco (RBC) promoter [39]. An LR reaction (Gateway-LR Clonase TM II) was carried out to clone each gene into pBinPlus binary [40] vector between the right and left borders of the T-DNA for plant transformation.

### Transient Expression in *N. benthamiana*

*A. tumefaciens* infiltration (agro-infiltration) was performed according to the description of van Herpen *et al.* [24]. *A. tumefaciens* batches were grown at 28°C at 220 rpm for 24 h in YEP media with kanamycin (50 mg/L) and rifampicillin (34 mg/L). Cells were harvested by centrifugation for 20 min at 4000×g and 20°C and then resuspended in 10 mM MES buffer containing 10 mM MgCl_2_ and 100 µM acetosyringone (4′-hydroxy-3′,5′-dimethoxyacetophenone, Sigma) to a final OD_600_ of *c.* 0.5, followed by incubation at room temperature under gentle shaking at 50 rpm for 150 min. For co-infiltration, equal volumes of the *A. tumefaciens* batches were mixed. Batch mixtures were infiltrated into leaves of three-week-old *N. benthamiana* plants by pressing a 1 mL syringe without metal needle against the abaxial side of the leaf and slowly injecting the bacterium suspension into the leaf. *N. benthamiana* plants were grown from seeds on soil in the greenhouse with a minimum of 16 h light. Day temperatures were approximately 28°C, night temperatures 25°C. After agro-infiltration the plants were grown under the same greenhouse conditions for another 3 days and then harvested for analysis.

### GC-MS Analysis of Yeast and Agro Infiltrated Plant Extracts

Seven mL yeast culture was extracted three times with 2 mL ethyl acetate, which was concentrated, dried using anhydrous Na_2_SO_4_ and used for GC-MS analysis. Agro-infiltrated leaves (100 mg) were ground in liquid nitrogen and extracted by brief vortexing and sonication for 10 min in 800 µl dichloromethane (DCM). The extracts were centrifuged for 15 min at 3000 rpm, dehydrated using Na_2_SO_4_ and then used for GC-MS analysis as described above. Compounds were identified by comparison of mass spectra and retention times (RT) with those of the authentic standards of germacrene A (GA), germacra-1(10),4,11(13)-trien-12-ol (GOH), germacra-1(10),4,11(13)-trien-12-al (GAL) [37] and costunolide (COS) (TOCRIS bioscience). Quantification of these sesquiterpenoids was conducted by determination of the total ion current (TIC) peak areas of the sesquiterpenoid peaks from three independent fermentation experiments. At the routine injection port temperature of 250°C germacrene A, germacra-1(10),4,11(13)-trien-12-oic acid (GAA) and costunolide are thermally converted into β-elemene, elematrien-12-oic acid, and saussurea lactone, respectively as discussed by de Kraker *et al.* [16], [18], [41]. We also regularly injected samples with an injection port temperature of 150°C to confirm the presence of non-rearranged germacrene A, germacra-1(10),4,11(13)-trien-12-oic acid and costunolide.

### LC-QTOF-MS Analysis

Non-volatile metabolites were analysed by LC–QTOF-MS (liquid chromatography, coupled to quadrupole time-of-flight mass spectrometry) according to a protocol for untargeted metabolomics of plant tissues [42]. A Waters Alliance 2795 HPLC connected to a Waters 2996 PDA detector and subsequently a QTOF Ultima V4.00.00 mass spectrometer (Waters, MS technologies, UK) operating in negative ionization mode was used. An analytical column (Luna 3 μ C18/2 100A; 2.0×150 mm) attached to a C18 pre-column (2.0×4 mm) (both from Phenomenex, USA) was used. Degassed eluent A [ultra-pure water: formic acid (1000∶1, v/v)] and eluent B [acetonitrile:formic acid (1000∶1, v/v)] were used at a flow rate of 0.19 mL min^−1^. Masses were recorded between m/z 60 and m/z 1000; leucine enkaphalin ([M-H]− = 554.2620) was used as a lock mass for on-line accurate mass correction. For agro-infiltrated *N. benthamiana*, 100 mg infiltrated leaf from each treatment was ground in liquid nitrogen and extracted with 300 µl methanol:formic acid (1000∶1, v/v). After brief vortexing and sonication for 15 min, the extracts were centrifuged for 5 min at 13,000 rpm and filtered through a 0.2 µm inorganic membrane filter (RC4, Sartorius, Germany). The gradient of the HPLC started at 5% eluent B and increased linearly to 75% eluent B in 45 min, after which the column was washed and equilibrated for 15 min before the next injection. The injection volume was 5 µl.

### Headspace Analysis and GC-MS Thermodesorption

Volatile collection from agro-infiltrated *N. benthamiana* leaves and GC-MS analysis were performed according to van Herpen *et al.* [24]. Steel sorbent cartridges (89 mm 429×6.4 mm O.D.; Markes) containing Tenax were used for volatile collection. Cartridges were conditioned at 280°C for 40 min under a nitrogen flow of 20 psi in a TC-20 multi-tube conditioner and were capped airtight until use. *N. benthamiana* leaves were detached and placed on water in a small vial and were enclosed in a glass container. To trap the leaf-produced volatiles, air was sucked through one Tenax cartridge (to purify the incoming air) and then through the containers and a second cartridge to adsorb volatiles at a flow rate of 90 mL/min for 24 h. Sample cartridges were dried for 15 min at room temperature with a nitrogen flow of 20 psi before GC-MS analysis on a Thermo Trace GC Ultra connected to a Thermo Trace DSQ quadruple mass spectrometer (Thermo Fisher Scientific, USA). Cartridges were placed in an automated thermodesorption unit (Ultra; Markes, Llantrisant) in which they were flushed with helium at 50 mL/min for 2 min to remove moisture and oxygen just before thermodesorption. The volatiles were desorbed by heating of the cartridges at 220°C for 5 min with a helium flow of 50 mL/min. The compounds released were trapped on an electrically cooled sorbent trap (Unity; Markes, Llantrisant) at a temperature of 5°C. Subsequently, the trapped volatiles were injected on the analytical column (ZB-5MSI, 30 m×0.25 mm ID, 1.0 µm film thickness, Zebron, Phenomenex) in splitless mode by ballistic heating of the cold trap to 250°C for 3 min. The temperature program of the GC started at 40°C (3 min hold) and rose 10°C/min to 280°C (2 min hold). The column effluent was ionised by electron impact (EI) ionisation at 70 eV. Mass scanning was done from 33 to 280 *m/z* with a scan time of 4.2 scans/sec. Xcalibur software (Thermo, USA) was used to identify the eluted compounds by comparing the mass spectra with those of authentic reference standards.

### Gene Expression Analysis

For RNA extraction plant tissue was homogenized by adding one pre-cooled grinding bead to each 2 mL Eppendorf vial containing 50–100 mg of liquid nitrogen frozen plant tissue and using a pre-cooled Mikro-disembrator II (Braun; Germany) for 1 min at maximum speed. After careful removal of the beads, RNA was isolated using TriPure (Roche) and transcribed into cDNA using TaqMan Reverse Transcription reagents (Applied Biosystems) according to the manufacturer’s instructions.

RT-qPCR was used to study the expression of *TcGAS*, *TcGAO*, *TcCOS* in cDNA derived from different tissues. Gene specific primers were design using Beacon Designer Software. *T. cinerariifolium* glyceraldehyde 3-phosphate dehydrogenase (GAPDH) (TcGAPDH-F: 5′- AGACGAGTTTCACAAAGTTG-3′ and TcGAPDH-R ‘5-AGGAATCTGAAGGCAAGC-3′) was used for normalization. PCR reactions were prepared in duplicate by mixing 22.5 µL iQ SYBR green supermix 2× (Biorad), 4.5 µL sense primer (3 µM), 4.5 µL antisense primer (3 µM), 11.5 µL deionized water, and 2 µL cDNA template in a 500 µL Eppendorf vial. After vortexing, 2×20 µL of each sample was distributed into two wells in 20 mL amounts. Quantification of the transcript level was performed in an MyiQ iCycler system (Bio-Rad Laboratories, USA) using a three-step programme, which included (i) enzyme-activation at 95°C for 3 min, (ii) 40 cycles of 95°C for 10 sec, 60°C for 30 sec, and (iii) 95°C for 1 min, from 65°C to 95°C for 10 sec for dissociation curve analysis. At the end of each run, amplified products were sequenced to verify their identity. Relative gene expression (RGE) values were calculated using the efficiency δCt method.

## Supporting Information

Figure S1 **Multiple protein sequence alignment of germacrene A synthase sequences.** Alignment based on the deduced amino acid sequence of pyrethrum germacrene A synthase (TcGAS, genebank: KC441526) and other characterized plant GASs. The alignment was performed using ClustalW2 (http://www.ebi.ac.uk/Tools/msa/clustalw2). The species abbreviations are Ci, *Cichorium intybus*; Ha, *Helianthus annuus*; Aa, *Artemisia annua*; Tp, *Tanacetum parthenium*.
(DOCX)

Figure S2 **Multiple protein sequence alignment of germacrene A oxidase sequences.** Alignment based on the deduced amino acid sequences of pyrethrum germacrene A oxidase (TcGAO, genebank: KC441527) and other characterized plant GAOs. The alignment was performed using ClustalW2 (http://www.ebi.ac.uk/Tools/msa/clustalw2). The species abbreviations are Ci, *Cichorium intybus*; Ha, *Helianthus annuus*.
(DOCX)

Figure S3 **Multiple protein sequence alignment of costunolide synthase sequences.** Alignment based on the deduced amino acid sequences of pyrethrum costunolide synthase (TcCOS, genebank: KC441528) and other characterized plant COSs. The alignment was performed using ClustalW2 (http://www.ebi.ac.uk/Tools/msa/clustalw2). The species abbreviations are Ci, *Cichorium intybus*; Ha, *Helianthus annuus*; *Ls, Lactuca sativa.*
(DOCX)

Table S1 **List of compounds detected with GC-MS on pyrethrum chloroform dip extracted seeds, with putative identification where possible.**
(DOCX)
