# Supplementary material for: Biosynthesis of Sesquiterpene Lactones in Pyrethrum (Tanacetum cinerariifolium)
Source: PLoS One. 2013 May 31;8(5):e65030. doi: 10.1371/journal.pone.0065030 (PMC3669400; doi:10.1371/journal.pone.0065030)
Supplement: Figure S1 — Multiple protein sequence alignment of germacrene A synthase sequences. Alignment based on the deduced amino acid sequence of pyrethrum germacrene A synthase (TcGAS, genebank: KC441526) and other characterized plant GASs. The alignment was performed using ClustalW2 (http://www.ebi.ac.uk/Tools/msa/clustalw2). The species abbreviations are Ci, Cichorium intybus; Ha, Helianthus annuus; Aa, Artemisia annua; Tp, Tanacetum parthenium. (DOCX) [file pone.0065030.s001.docx]

**
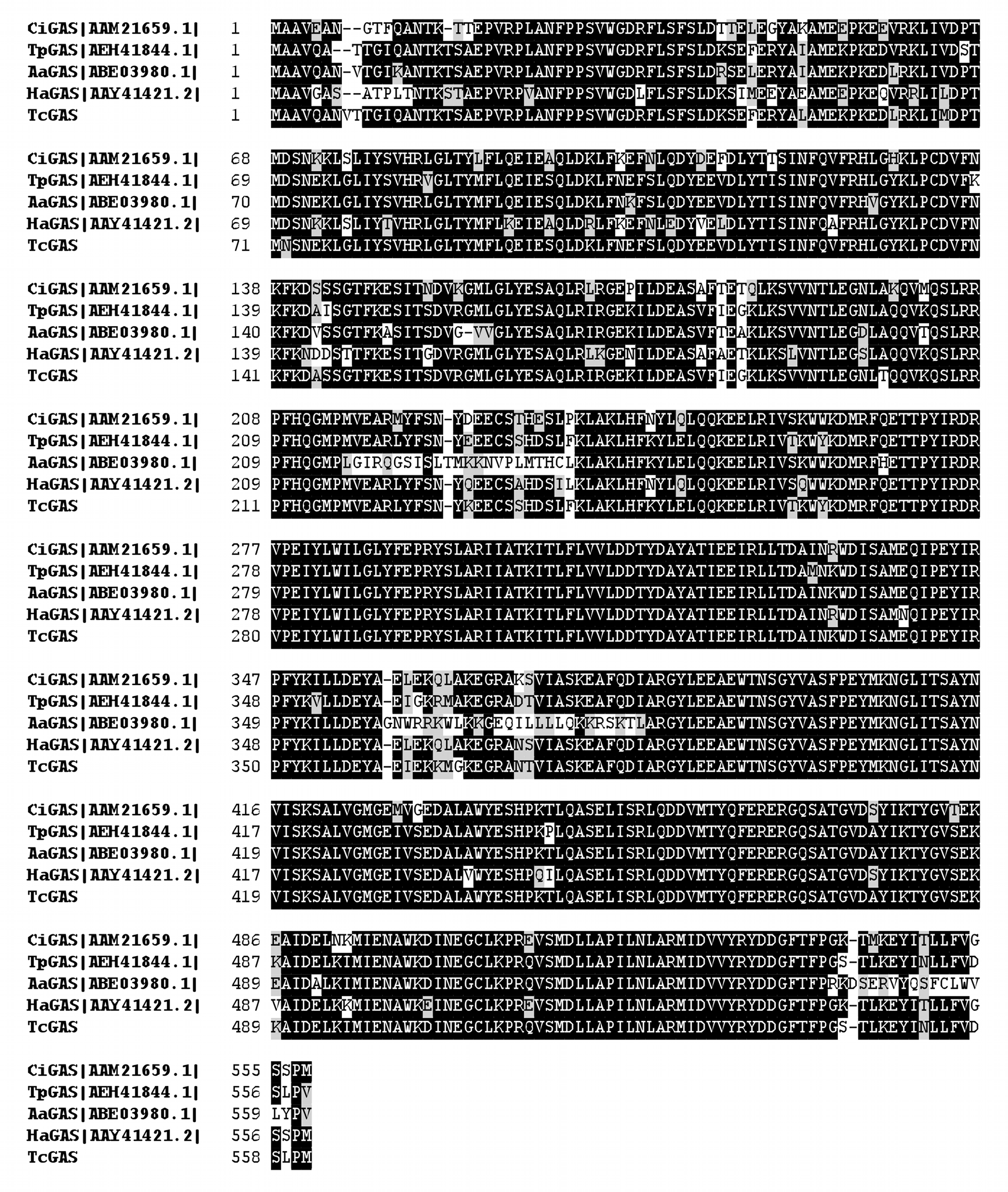
**

**Figure S1. Multiple protein sequence alignment of germacrene A synthase sequences.** Alignment based on the deduced amino acid sequence of pyrethrum germacrene A synthase (TcGAS, genebank: KC441526) and other characterized plant GASs. The alignment was performed using ClustalW2 (http://www.ebi.ac.uk/Tools/msa/clustalw2). The species abbreviations are Ci, *Cichorium intybus*; Ha, *Helianthus annuus*; Aa, *Artemisia annua*; Tp, *Tanacetum parthenium*.
