# Supplementary material for: Biosynthesis of Sesquiterpene Lactones in Pyrethrum (Tanacetum cinerariifolium)
Source: PLoS One. 2013 May 31;8(5):e65030. doi: 10.1371/journal.pone.0065030 (PMC3669400; doi:10.1371/journal.pone.0065030)
Supplement: Figure S3 — Multiple protein sequence alignment of costunolide synthase sequences. Alignment based on the deduced amino acid sequences of pyrethrum costunolide synthase (TcCOS, genebank: KC441528) and other characterized plant COSs. The alignment was performed using ClustalW2 (http://www.ebi.ac.uk/Tools/msa/clustalw2). The species abbreviations are Ci, Cichorium intybus; Ha, Helianthus annuus; Ls, Lactuca sativa. (DOCX) [file pone.0065030.s003.docx]

**
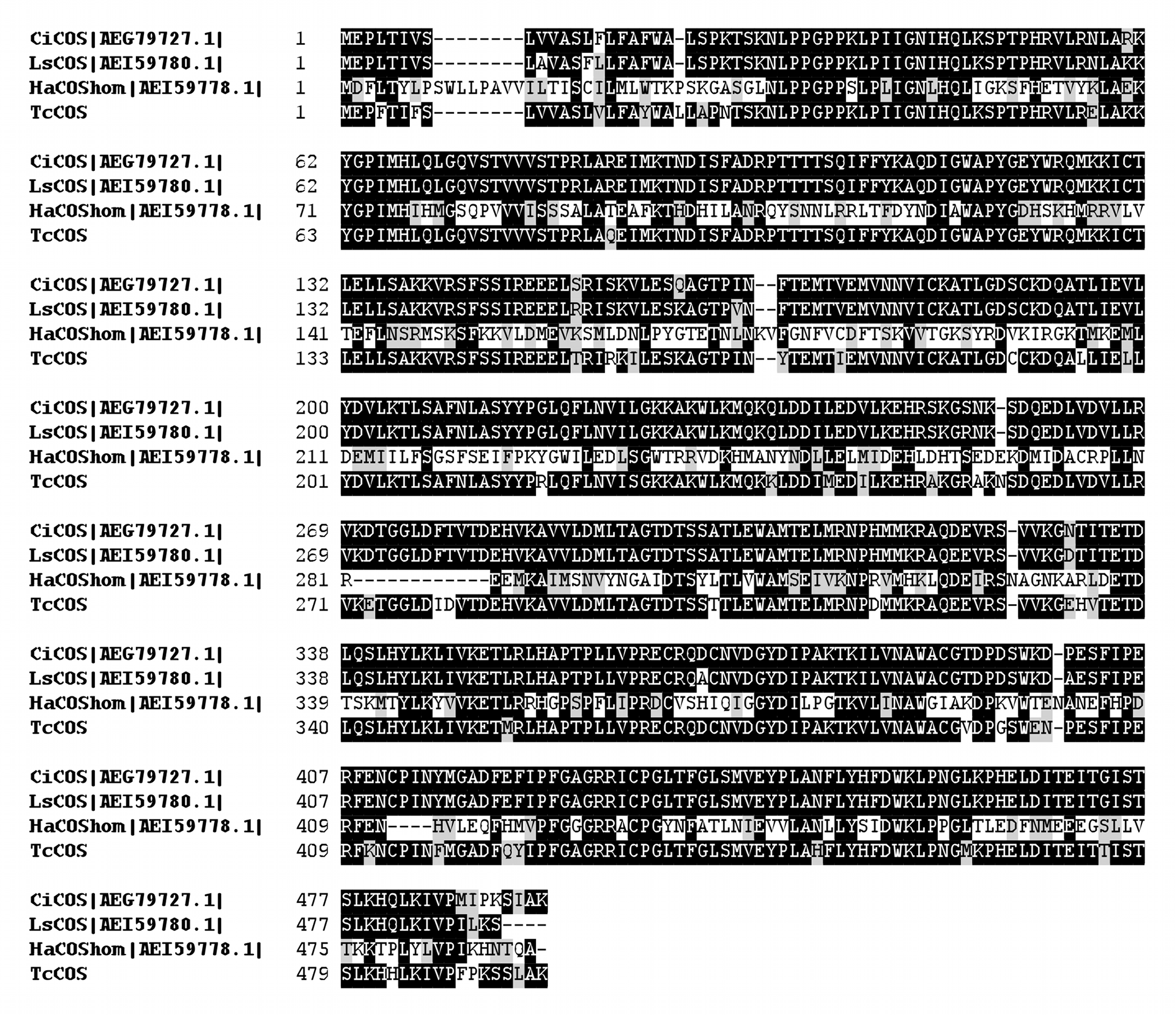
**

**Figure S3. Multiple protein sequence alignment of costunolide synthase sequences.** Alignment based on the deduced amino acid sequences of pyrethrum costunolide synthase (TcCOS, genebank: KC441528) and other characterized plant COSs. The alignment was performed using ClustalW2 (http://www.ebi.ac.uk/Tools/msa/clustalw2). The species abbreviations are Ci, *Cichorium intybus*; Ha, *Helianthus annuus*; *Ls, Lactuca sativa.*
