# Supplementary material for: Biosynthesis of Sesquiterpene Lactones in Pyrethrum (Tanacetum cinerariifolium)
Source: PLoS One. 2013 May 31;8(5):e65030. doi: 10.1371/journal.pone.0065030 (PMC3669400; doi:10.1371/journal.pone.0065030)
Supplement: Table S1 — List of compounds detected with GC-MS on pyrethrum chloroform dip extracted seeds, with putative identification where possible. (DOCX) [file pone.0065030.s004.docx]

**Table S1. List of compounds detected with GC-MS on pyrethrum chloroform dip extracted seeds, with putative identification where possible**

**
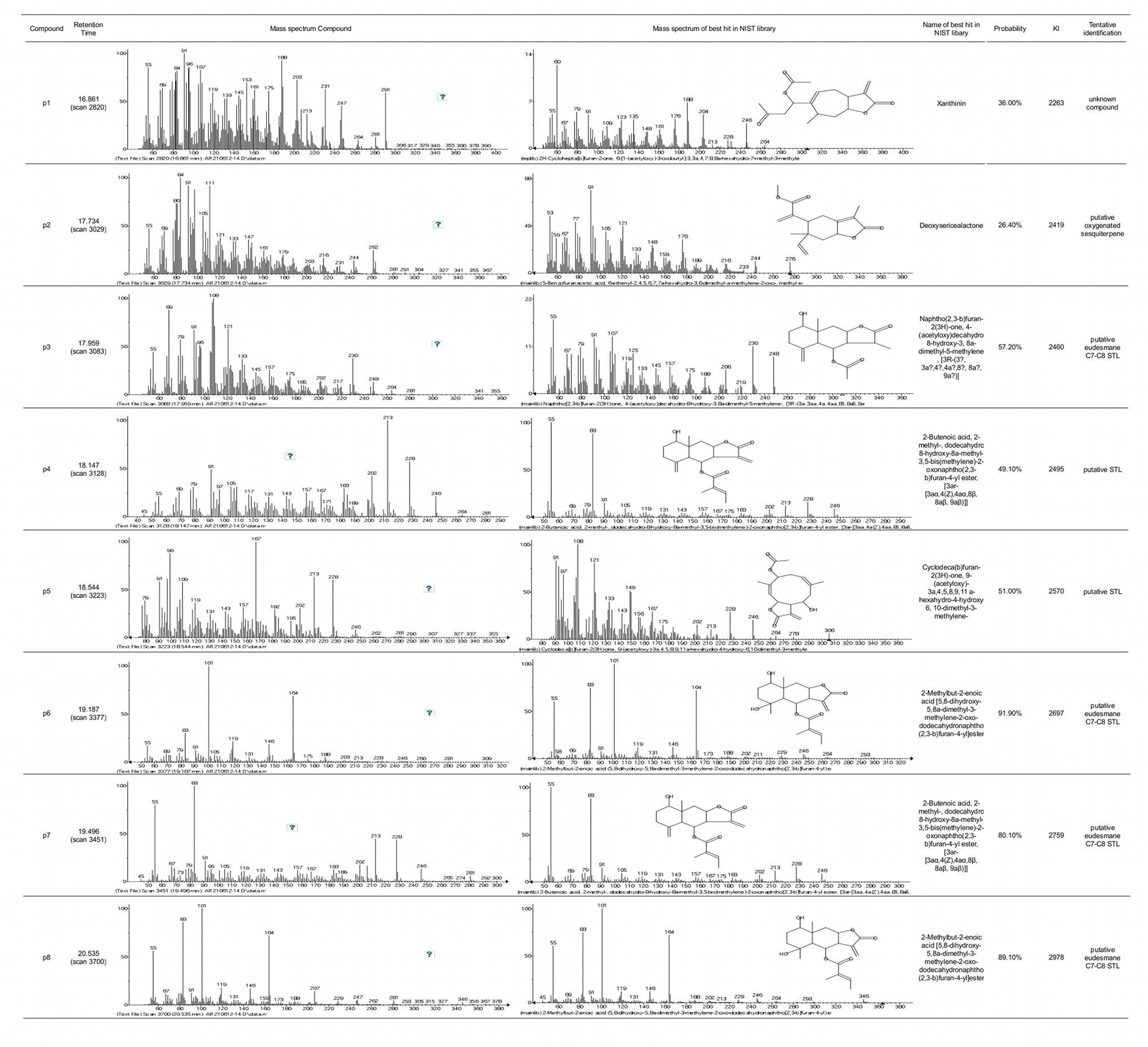
**
